# An adaptive acoustoelectric signal decoding algorithm based on Fourier fitting for brain function imaging

**DOI:** 10.3389/fphys.2022.1054103

**Published:** 2022-12-08

**Authors:** Xizi Song, Tong Wang, Mengyue Su, Xinrui Chen, Xiuyun Liu, Dong Ming

**Affiliations:** ^1^ Academy of Medical Engineering and Translational Medicine, Tianjin University, Tianjin, China; ^2^ Department of Biomedical Engineering, College of Precision Instruments and Optoelectronics Engineering, Tianjin University, Tianjin, China

**Keywords:** acoustoelectric brain imaging, decoding algorithm, Fourier fitting, acoustoelectric effect, brain function imaging

## Abstract

Acousticelectric brain imaging (ABI), which is based on the acoustoelectric (AE) effect, is a potential brain function imaging method for mapping brain electrical activity with high temporal and spatial resolution. To further enhance the quality of the decoded signal and the resolution of the ABI, the decoding accuracy of the AE signal is essential. An adaptive decoding algorithm based on Fourier fitting (aDAF) is suggested to increase the AE signal decoding precision. The envelope of the AE signal is first split into a number of harmonics by Fourier fitting in the suggested aDAF. The least square method is then utilized to adaptively select the greatest harmonic component. Several phantom experiments are implemented to assess the performance of the aDAF, including 1-source with various frequencies, multiple-source with various frequencies and amplitudes, and multiple-source with various distributions. Imaging resolution and decoded signal quality are quantitatively evaluated. According to the results of the decoding experiments, the decoded signal amplitude accuracy has risen by 11.39% when compared to the decoding algorithm with envelope (DAE). The correlation coefficient between the source signal and the decoded timing signal of aDAF is, on average, 34.76% better than it was for DAE. Finally, the results of the imaging experiment show that aDAF has superior imaging quality than DAE, with signal-to noise ratio (SNR) improved by 23.32% and spatial resolution increased by 50%. According to the experiments, the proposed aDAF increased AE signal decoding accuracy, which is vital for future research and applications related to ABI.

## 1 Introduction

The monitoring of neurological conditions such as epilepsy especially for neuro-intensive care, depression, and brain-computer interface (BCI) control using brain spontaneously or evokedly electrical activity is prevalent (Cao et al., 2018; Xu et al., 2018; Panwar et al., 2019). Since 1924, electroencephalography (EEG), which uses a hundred or fewer electrodes to measure electric potentials generated by the brain on the scalp surface, has been used to detect brain electrical activity (Grover and Venkatesh 2016). Voltage is recorded using incredibly sensitive surface electrodes. EEG has a constrained spatial resolution, though. High temporal and spatial resolution non-invasive imaging of electrical activity is still difficult to accomplish. Confirm brain electrical activity, high-density electroencephalography (hdEEG), for instance, with 256 electrodes, is a supplementary and non-invasive neurophysiology method. The aim of electrical source imaging is to locate the sources of scalp EEG signals within the brain by resolving an ill-posed inverse problem. Furthermore, hdEEG is still fundamentally constrained by an inadequate spatial resolution (Hedrich et al., 2017). It is essential to develop a method with high performance that enables direct mapping of electrical activation in order to better comprehend these brain disorders, make more accurate diagnoses, and operate BCI with precision.

With high temporal and spatial resolution, acoustoelectric (AE) imaging, which is based on the AE effect, has the ability to map biological current densities directly. Focused ultrasound can change the resistivity at the focal spot leading to the AE effect, a type of physical interaction between the acoustic and electrical fields. For electrolyte solution (Fox et al., 1946; Jossinet et al., 1998; Jossinet et al., 1999; Lavandier et al., 2000a; Lavandier et al., 2000b; Li et al., 2012) and tissue (Song et al., 2017) respectively, its mechanisms have been studied. Bench-top experiments have demonstrated the viability of using AE imaging to map biological current Wang et al., 2011, and the parameters, such as frequency and pulse shape, are optimized (Qin et al., 2012). Besides that, heart is used *in vivo* investigations (Qin et al., 2015; Berthon et al., 2019). And, a highly sensitive ultrafast AE imaging system is included (Berthon et al., 2017). A potential application for AE imaging, subsequently, is the selective mapping of lead currents using DBS device (Preston et al., 2018). In 2017, the concept of Acoustoelectric brain imaging (ABI) was introduced, which expressly refers to the measurement of brain electrical signal *via* ABI. A pulse repetition frequency (PRF) coding technique for ABI is be researched in 2020 (Zhou et al., 2020), then.

The AE signal, which retrieved from recored EEG signal, is directly proportional to current density and sensitive to the direction of current flow. Therefore, the AE signal could be used to reconstruct the current source density image, and then realize the reconstruction of the activation source in the human brain. Time-space resolution in ABI result is affected derectly by the decoding accuracy of AE signals. Because ABI is a novel brain imaging technique, the decoding algorithm used in it is still envelope algorithm based on Hilbert transform. The basic work of early AE imaging decoded with envelope algorithm (Song et al., 2019; Zhou et al., 2019). Zhou et al., in 2020, conducted the first multi-source acoustoelectric imaging experiment (Zhou et al., 2020), which adopted algorithm based on the envelope function to decode the AE signal. For the first time, ABI decoded the Steady state visual evoked signal in the brain of a living mouse and also exploited the envelope algorithm to decode the AE signal (Song et al., 2021). Since the brain contains a sea of neurons that fire simultaneously, the frequency and amplitude properties of these many current sources are complex, the current decoding algorithm for the AE signal needs to be optimized and improved to improve imaging quality for practical application.

In this experiment, we designed a recent algorithm, an adaptive decoding approach based on Fourier fitting (aDAF), to improve the decoding accuracy of the AE signal, and verified the feasibility of the algorithm in the phantom experiment and then compared the performance of the classic algorithm (DAE). In addition, imaging experiments claim that the new algorithm markedly improves the spatial resolution of the ABI. The comprehensive performance demonstrates the innovation and superior performance of the aDAF in ABI decoding. Therefore, the application potential of ABI in the field of clinical diagnosis and brain-computer interface is enhanced.

## 2 Materials and methods

### 2.1 Acoustoelectric brain imaging

Based on AE effect, ABI is a novel neuroimaging approach. When ultrasound travels through biological tissue, the conductivity modulation brought on by acoustic pressure. The conductivity modulation by acoustic waves were calculated as follows:

$$\begin{array}{c} \frac{1}{∆\sigma }=\frac{1}{\sigma_{0}}K∆P \end{array}$$ (1)

Where $\sigma_{0}$ is the initial conductivity and ∆σ is the changed conductivity, ∆P is the acoustic pressure and then K is a constant of interaction that value is on the order of 10^–9^ Pa^−1^ in a 0.9% NaCl solution (Jossinet et al., 1998). A lead is a group of two electrodes. Lead field refers to the sensitivity distribution of a lead. The lead field and the current flow field that results from applying a unit current to the lead are identical (Malmivuo and Plonsey, 1995; Song et al., 2019; Zhou et al., 2019; Zhou et al., 2021). According to lead field theory, the voltage $V_{i}$ measured by lead i is:

$$\begin{array}{c} V_{i}=∭\frac{1}{\sigma }\left({J_{i}}^{L}\cdot J^{I}\right)dxdydz \end{array}$$ (2)

Where ${J_{i}}^{L}$ represents the lead field and $J^{I}=J^{I}\left(x,y,z\right)$ represents source current density.

The distribution of conductivity changes when an ultrasonic wave travels through biological tissue, and the resulting conductivity distribution is:

$$\begin{array}{c} \frac{1}{\sigma }=\frac{1}{\sigma_{0}}+\frac{1}{\sigma_{0}}K\Delta P \end{array}$$ (3)

Substituting (3) into (2), the resulting voltage satisfies:

$$\begin{array}{c} V_{i}={V_{i}}^{AE}+{V_{i}}^{LF} \end{array}$$ (4)

$$\begin{array}{c} {V_{i}}^{LF}=\frac{1}{\sigma_{0}}\left({J_{i}}^{L}\cdot J^{I}\right)dxdydz \end{array}$$ (5)

$$\begin{array}{c} {V_{i}}^{AE}=\frac{1}{\sigma_{0}}K\Delta P\left({J_{i}}^{L}\cdot J^{I}\right)dxdydz \end{array}$$ (6)

Where ${V_{i}}^{LF}$ represents the low-frequency (DC-10 kHz) content of $V_{i}$ while ${V_{i}}^{AE}$ represents the high-frequency (MHz) AE signal (Olafsson et al., 2008).

The AE signal at the focal point can be derived, according to (6). Since the location of the focal point is determined, millimeter-focused ultrasound can sift an AE signal having good spatial resolution and positional accuracy. ABI can create images with spatial resolution limited to the ultrasound focus after scanning the relevant areas of the brain and collecting the decoded AE signal.

### 2.2 Decoding algorithm with envelope

In the research of ABI, the Decoding Algorithm with Envelope is frequently utilized (Qin et al., 2012; Qin et al., 2015; Berthon et al., 2017; Preston et al., 2018; Berthon et al., 2019; Zhou et al., 2020; Zhou et al., 2021). The theory (Zhou et al., 2019) stipulates that the AE signal is first converted into an analytical signal. In the construction of analytic signal, the high frequency components of the actually recorded signal were taken as the real part, and the result from which the real part have been done Hilbert transform were taken as the imaginary part.

$$\begin{array}{c} \tilde{x\left(t\right)}=x\left(t\right)+j\hat{x\left(t\right)} \end{array}$$ (7)

where $\tilde{x\left(t\right)}$ is the constructed analytic signal from the AE signal, $x\left(t\right)$ is AE signal, $\hat{x\left(t\right)}$ is the result from which the AE signal have been done Hilbert transform. The AE signal contains both amplitude and phase information. And it can be expressed as:

$$\begin{array}{c} x\left(t\right)=A\left(t\right)\cos\left(\omega_{0}+\theta \left(t\right)\right) \end{array}$$ (8)

Substituting (8) into (7) leads to:

$$\begin{array}{c} \tilde{x\left(t\right)}=A\left(t\right)\cos\left(\omega_{0}+\theta \left(t\right)\right)+jA\left(t\right)\sin\left(\omega_{0}+\theta \left(t\right)\right) \end{array}$$ (9)

$$\begin{array}{c} \left|\tilde{x\left(t\right)}\right|=\left|A\left(t\right)\right| \end{array}$$ (10)

Where $\left|A\left(t\right)\right|$ is the envelope of the AE signal, and the decoding result of DAE.

### 2.3 Adaptive decoding algorithm based on Fourier fitting

Towards the aim of further extract the frequency and amplitude characteristics of the source signal, an adaptive decoding algorithm based on Fourier fitting (aDAF) is developed. The aDAF includes two steps. Firstly, the envelope of AE signal is divided into multiple harmonics by Fourier fitting. Then, Harmonic component is adaptively selected by the least square method, which is the largest contribution to envelope.

#### 2.3.1 Fourier fitting

Fourier fitting can get the frequency characteristics of the original signal in time sequence. Any periodic function can be expanded into an infinite series of trigonometric functions (Guillén et al., 2007; Smetanin et al., 2020). The envelope signal contains periodic information of low frequency source signal. And the envelope signal $\left|A\left(t\right)\right|$ can be expanded by trigonometric function. Taking n as sample length, $\left|A\left(t\right)\right|$ can be extended to $\left(-\infty ,+\infty \right)$ . In interval $\left[-\frac{n}{2},\frac{n}{2}\right]$ , the fitting value of $\left|A\left(t\right)\right|$ , $\left|{A\left(t\right)}_{1}\right|$ can be expressed as the sum of a series of harmonics whose frequency is multiplied (Gonzalez et al., 2002), and the number is not more than $\frac{n}{2}$ .

$$\begin{array}{c} \left|A{\left(t\right)}_{1}\right|=a_{0}+\overset{m}{\underset{i=1}{\sum }}\mu_{i}\sin\left(iw_{0}t+\theta_{i}\right) \end{array}$$ (11)

Where $a_{0}$ is the DC component, and $m\;\left(\;m=\mathrm{int}\frac{n}{2}\;\right)$ represents the harmonic number. $\mu_{i}$ is twice the amplitude of each harmonic component. $\omega_{0}$ is the fundamental frequency of each harmonic ( $\omega_{0}=\frac{2\pi }{n}$ ). $\theta_{i}$ is the initial phase angle of each fundamental frequency. Expand formula 11 to get formula 12:

$$\begin{array}{c} \left|A{\left(t\right)}_{1}\right|=\overset{m}{\underset{i=0}{\sum }}a_{i}cosi\frac{2\pi }{n}t+b_{i}sini\frac{2\pi }{n}t \end{array}$$ (12)

Where $a_{i}\;\left(\;a_{i}=\mu_{i}\cos\theta_{i\;}\right)$ is the amplitude of each harmonic cosine component, and $b_{i}\;\left({\;b}_{i}=\mu_{i}\sin\theta_{i}\;\right)$ is the amplitude of each harmonic sinusoidal component.

#### 2.3.2 Least square method

The least square method is a mathematical optimization technique (Björck et al., 1990). It finds the best functional match of data by minimizing the square sum of errors. Using the least square method to determine the coefficients of $\left|{A\left(t\right)}_{1}\right|$ :

$$\begin{array}{c} \overset{n}{\underset{i=t}{\sum }}{\left|e_{i}\right|}^{2}=\overset{n}{\underset{i=t}{\sum }}{\left|A\left(t\right)-A{\left(t\right)}_{1}\right|}^{2} \end{array}$$ (13)

Where $e_{i}$ is the fitting error. Minimizing the sum of squares of $e_{i}$ in Eq. 13.

Taking the $\overset{n}{\underset{i=t}{\sum }}{\left|e_{i}\right|}^{2}$ partial derivative of $a_{i}$ and $b_{i}$ respectively, and setting the value of the partial derivative to zero. Using the orthogonality of trigonometric function. The following results can be obtained:

$$\begin{array}{c} a_{0}=\frac{1}{n}\overset{n}{\underset{i=1}{\sum }}\left|A\left(t\right)\right| \end{array}$$ (14)

$$\begin{array}{c} a_{i}=\frac{2}{n}\overset{n}{\underset{i=1}{\sum }}\left|A\left(t\right)\right|\cos i\frac{2\pi }{n}t \end{array}$$ (15)

$$\begin{array}{c} b_{i}=\frac{2}{n}\overset{n}{\underset{i=1}{\sum }}\left|A\left(t\right)\right|\sin i\frac{2\pi }{n}t \end{array}$$ (16)

$$\begin{array}{c} c_{i}^{2}=a_{i}^{2}+b_{i}^{2} \end{array}$$ (17)

### 2.4 Experimental device setup

**FIGURE 1:**
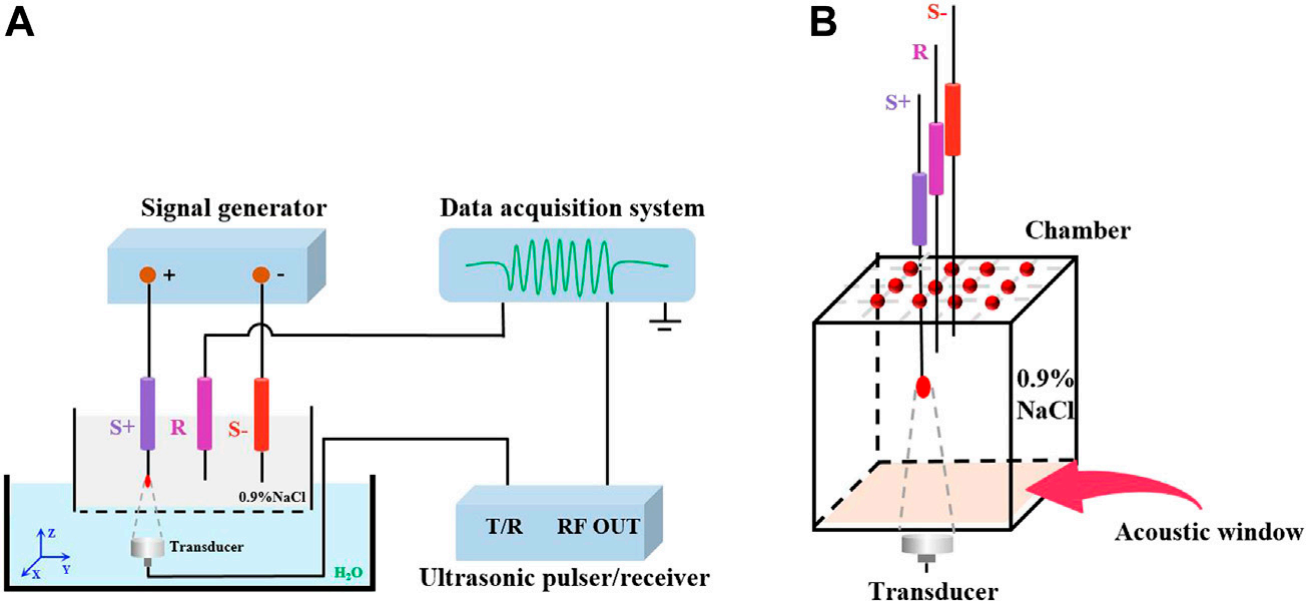
**(A)** Schematic of experimental setup. **(B)** Detail of the chamber.

The experimental apparatus applied in this study is shown in Figure 1. The chamber was repleted with 0.9% NaCl solution. As an acoustic window, a 3M Transparent Film (Tegaderm™, United States) was fastened to the base of the chamber. According to Figure 1A, the triggering device is an ultrasonic pulser/receiver (Olympus model 5077P. JP) with a repetition rate of 1 kHz. An ultrasonic transducer (Olympus, A392S, 63.5 mm focal length) with a single element operating at 1 MHz generates focused ultrasound. An electronic signal generator (RIGOL DG4162) produces the current source for research. The *z*-axis is the direction in which an ultrasonic wave travels, and the x-y plane is mechanically scanned by the ultrasonic transducer. With the purpose of giving the rise to a dipole field, 0.9% NaCl solution was poured over two platinum electrodes (S+, S-). The recording electrode (R) was employed to detect the AE signal. The AE signal was acquired and amplified by SynAmps2 system (Neuroscan, United States), and sampling rate was 20 kHz.

### 2.5 Experimental design

Three experiments with various features, including a single source with various frequencies, a pair of sources with various frequencies and amplitudes, and a three source with various frequencies were implemented for the experiment of decoding performance evaluation.

#### 2.5.1 1-Source with different frequencies

**FIGURE 2:**
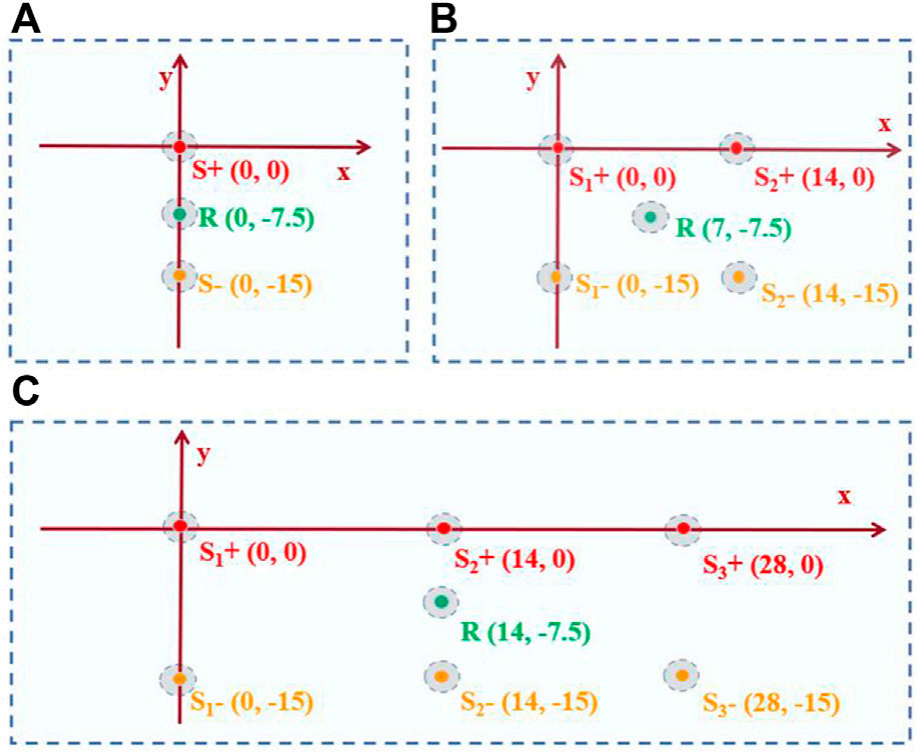
Experimental design for decoding performance evaluation. **(A)** Is the 1-source diagram. **(B)** is the 2-source diagram. **(C)** is the 3-source diagram. S+ is the positive electrode placement position. R is the recording electrode placement position. S- is the negative electrode placement position.

The diagram for the 1-source experiment is shown in Figure 2A. A pair of stimulation electrodes were submerged in saline for the 1-source experiment. The anode source is the precise focus of the ultrasonic transducer. Three groups of 1-source studies at various frequencies, such as 8 Hz, 10 Hz, and 13 Hz, were conducted. Along with that, matching AE signals are measured.

#### 2.5.2 2-Source with different frequencies and amplitudes

The diagram for the 2-source experiment is shown in Figure 2B. In saline water, two sets of stimulation electrodes were submerged. To measure the AE signal, the focused ultrasound is first focused on one anode, S1+. The concentrated ultrasound is then focused on anode S2+, where the AE signal is measured. The frequencies of S1+ and S2+ were set in saline water for the experiment with two sources with different frequencies and the same amplitude (100 mV). Two groups of experiments with the same frequency were created for the experiment with two sources of varying amplitudes (10 Hz). The signals amplitudes of two source in the first group experiment were 100 mV (S1+) and 50 mV (S2+), respectively. In another group experiment, the amplitudes of the two source signals were 150 mV (S1+) and 50 mV (S2+), respectively.

#### 2.5.3 3-Source with different frequencies

The 3-source experiment diagram is depicted in Figure 2C. In saline, three sets of stimulation electrodes were submerged. Three anodes (S1+, S2+, and S3+) were focused on by focused ultrasound, which measures the corresponding AE signal. The frequencies of S1+, S2+, and S3+ were 7 Hz, 10 Hz, and 13 Hz, respectively, with the same amplitude (100 mV).

Imaging experiments, including 2-source and 3-source imaging experiments, were carried out to test the decoding performance further.

#### 2.5.4 2-Source imaging experiment

**FIGURE 3:**
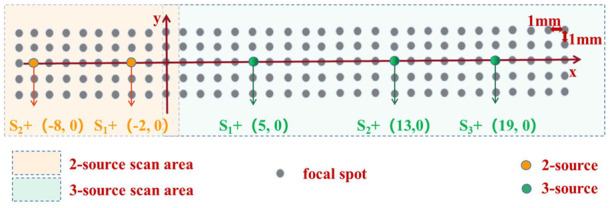
Experimental design for the imaging experiment. S+ represents the placement position of positive electrodes. Gray represents the focal spot of ultrasound. The yellow area indicates 2-source scanning point area. And the green area indicates 3-source scanning point area.

The 2-source scanning zone is the yellow area (10 × 5 focal spots) in Figure 3. The ultrasonic transducer scanned a distance of 9 mm (x = −9 mm ∼ 0 mm) along the *x* direction in steps of 1 mm, generating a total of 10 focus spots. It scanned 4 mm in the *y* direction (y = −2 mm ∼ 2 mm), producing a total of 5 focal points. Anode sources S1+ (−8 mm, 0 mm) and S2+ (−2 mm, 0 mm) were separated by 6 mm. The recording electrode was situated halfway between the two anode sources (−5 mm, −7.5 mm). Not in the scanning area were the corresponding cathodes S1- (−8 mm, −15 mm), and S2- (−2 mm, -15 mm). The sources had an amplitude of 100 mV and a frequency of 10 Hz.

#### 2.5.5 3-Source imaging experiment

The 3-source scanning region is shown by the green area (24 × 5 focal spots) in Figure 3. The transducer scanned an area of 23 mm in the *x* direction (x = 0 mm–23 mm), resulting in a total of 24 focus points. And it scanned 4 mm in the *y* direction (y = 2 mm–2 mm), producing a total of 5 focal points. Anode sources S1+ (5 mm, 0 mm) and S2+ (13 mm, 0 mm) were separated by 8 mm. Furthermore, there was a 6 mm spacing between anode sources S2+ (13 mm, 0 mm) and S3+ (19 mm, 0 mm). The three anode sources were arranged in a triangle, with the recording electrode (12 mm, −7.5 mm) in the center. S1- (5 mm, -15 mm), S2- (13 mm, -15 mm), and S3- (19 mm, −15 mm) cathodes, respectively, were not in the scanning area. The sources had an amplitude of 50 mV and a frequency of 10 Hz.

### 2.6 Signal processing

The obtained original signal was, first, down sampled at a rate of 5 KHz. After that, a third-order band-pass filter was exploited to filter the down sampled signal, the filtering range was between PRF-30 Hz and PRF+30 Hz. The filtered AE signal was decoded by applying DAE and aDAF to compare the performance the two had. The associated matrix of the decoded signal was interpolated at 0.01 to reconstruct the ABI in accordance with the known focal point coordinate. Decibels were put to use in the signal-to-noise ratio (SNR) calculation. The following is an expression for the SNR calculation formula:

$$\begin{array}{c} SNR=10\log_{10}\left(\frac{signal}{noise}\right) \end{array}$$ (18)

## 3 Results

### 3.1 Decoding results

#### 3.1.1 1-Source with different frequencies

**FIGURE 4:**
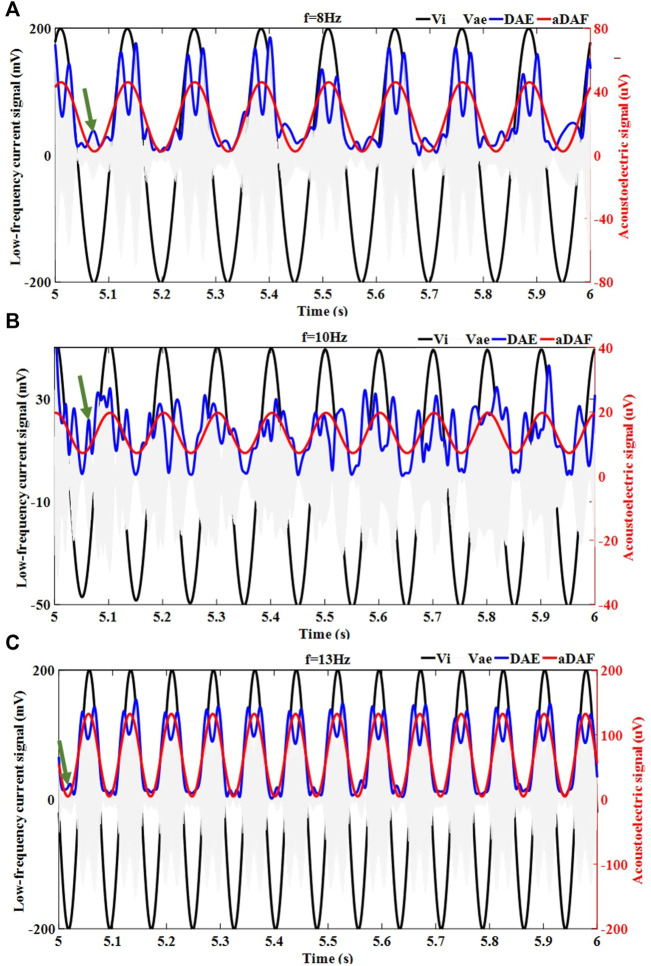
The decoded timing signal results for the 1-source with different frequencies, including **(A)** 8 Hz, **(B)** 10 Hz and **(C)** 13 Hz. The injected low frequency current signal ( $V_{i}$ ). Detected AE signal ( $V_{ae}$ ) with an amplifier. Decoded result of DAE (DAE). Decoded result of aDAF (aDAF).

Figure 4 displays the results of the decoded timing signal from 1-source at various frequencies (8 Hz, 10 Hz, and 13 Hz). It could be determined that the decoded timing signal of the aDAF contained the same frequency and phase information as the source signal for the 1-source with 8 Hz (Figure 4A). Additionally, the source signal had a positive correlation with the corresponding amplitude. The DAE decoded result were represented by the blue curve. The source signal and the decoded timing signal of DAE exhibited a positive correlation amplitude characteristic of the overall trend. But, the decoded timing signal fluctuated, and its frequency and phase were different from those of the source signal, as indicated by the green arrows. The correlation coefficient here between the source signal and the decoded result of the aDAF is 0.99 in digits. And there was a 0.79 correlation coefficient between the original signal and the decoded DAE result. The correlation coefficient of aDAF raised by 25.31% when compared to DAE. It was plain that aDAF was more efficient for decoding AE signals.

The results of 1-source with 10 Hz and 13 Hz decoding served to further substantiate the findings. It was seen in Figures 4B,C, the decoded timing signal of aDAF could characterize the frequency, phase, and amplitude details of the source signal more precisely than DAE. The correlation coefficients of aDAF and DAE for the single source at 10 Hz were 0.99 and 0.62, respectively. The correlation coefficients of aDAF and DAE for the single source at 13 Hz are 0.99 and 0.83, in both. So, the average correlation coefficient of aDAF for the three frequencies was 34.76% stronger than that of DAE. The results of this experiment confirmed that aDAF could extract the valid features of the source signal more efficiently than DAE.

#### 3.1.2 Multi-source with different frequencies

**FIGURE 5:**
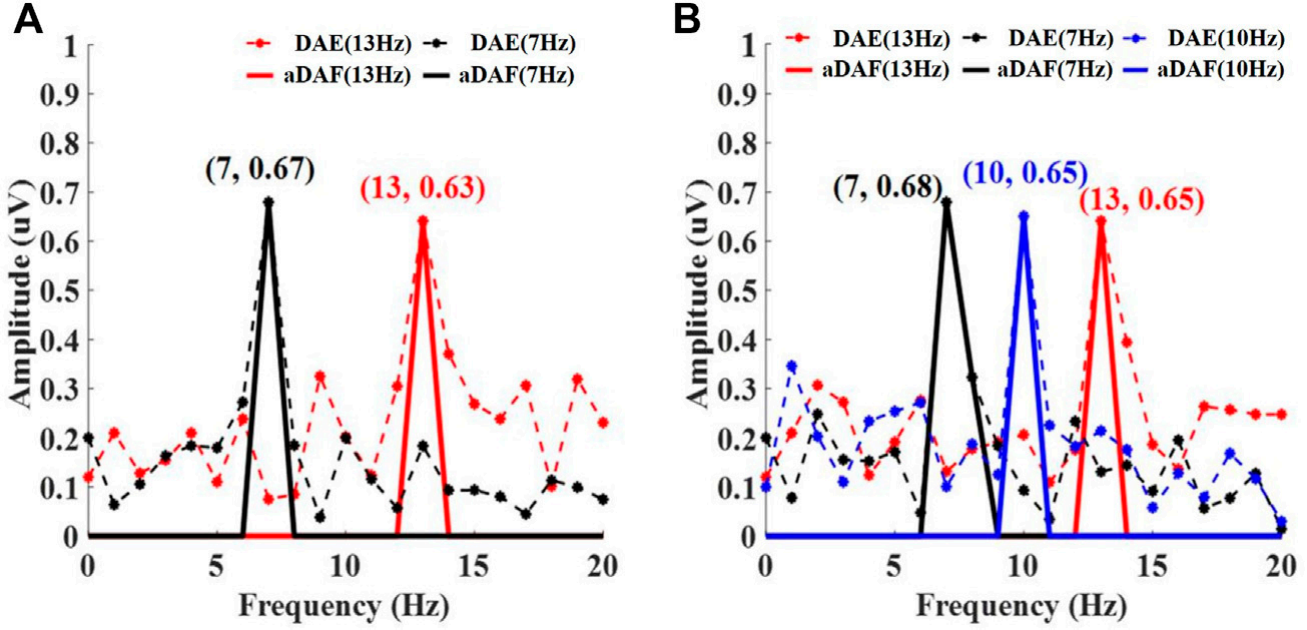
The decoded frequency spectrum results for multi-source with different frequencies, including **(A)** 2-source and **(B)** 3-source. In **(A)**, Black and red curve represent the spectrum corresponding to S1+ (7 Hz) and S2+ (13 Hz) respectively. In **(B)**, Black, blue and red curve represent the spectrum corresponding to S1+ (7 Hz), S2+ (10 Hz) and S3+ (13 Hz), respectively.

The frequency spectrum, which was determinded by adopting FFT, of the decoded results for the multiple sources with various frequencies was shown in Figure 5. Figures 5A,B respectively displays the findings of the 2-source and 3-source experiments. The results of the aDAF were shown by the solid line. And the decoded DAE findings were shown by the dotted line. The black curve in Figure 5A displayed the frequency spectrum related to S1+ (7 Hz). It was evident that the decoded signal for both aDAF and DAE exhibited an apparent high amplitude response at 7 Hz, which was compatible with the frequency of S1+. The frequency spectrum related to S2+ is shown by the red curve (13 Hz). The decoded signal of aDAF and DAE had a proper high amplitude response in the frequency range of 13 Hz, which was consistent with the frequency of S2+. At the same time, it was clear that for aDAF, the high amplitude response only arised at the frequency of the source signal and that its amplitude was nearly zero at the frequency of the non-source signal. Although the source signal frequency for DAE had the strongest amplitude response, there was amplitude response interference at the non-source signal frequency. In contrast to DAE, the decoded result of aDAF had a frequency spectrum that was devoid of interference and could accurately retrieve the frequency information of the source signal.

A 3-source experiment was implemented to further validate the aDAF performance. The high amplitude responses for DAE and aDAF were both visible at the relevant source signal frequency, as shown in Figure 5B. While the spectrum result of DAE is impacted by other non-source signal frequencies, the spectrum result of aDAF was almost totally unaffected by interference. Results from trials using multiple sources at various frequencies showed that aDAF can distinguish the frequency feature more clearly than DAE.

#### 3.1.3 Multi-source with different amplitudes

Two groups of multi-source experiments with various amplitudes are carried out to further examine the performance of aDAF. The amplitude ratio (V1/V2) for the first group was set at 2. And the decoded signal from DAE had an amplitude ratio of 1.60, while from aDAF it was 1.82. The amplitude accuracy of the decoded signal from aDAF was 13.75% better than that of DAE. The amplitude ratio (V1/V2) was then adjusted to 3 for the second group. DAE decoded the signal at an amplitude ratio of 3.32, while aDAF decoded the signal at an amplitude ratio of 3.02. The decoded signal of aDAF had an improved amplitude accuracy of 9.03% over DAE. The results of multi-source studies with various amplitudes show that aDAF could decode the amplitude characteristic more aptly. When compared to DAE, aDAF provides an average increase in amplitude accuracy of 11.39%.

### 3.2 Imaging results

#### 3.2.1 2-Source imaging result

**FIGURE 6:**
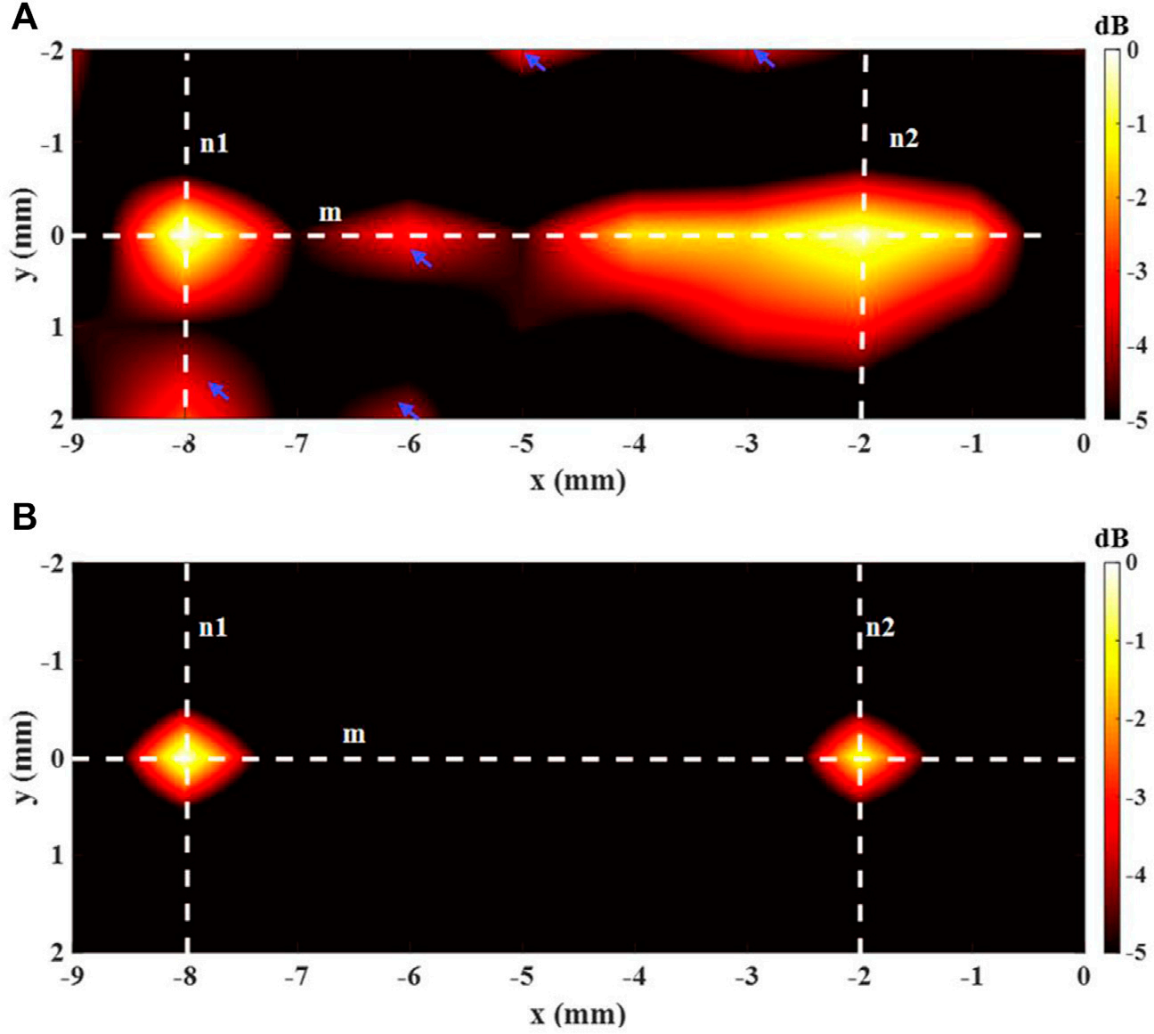
Imaging result of the 2-source with **(A)** DAE and **(B)** aDAF (hot colors, 5-dB dynamic range).

**FIGURE 7:**
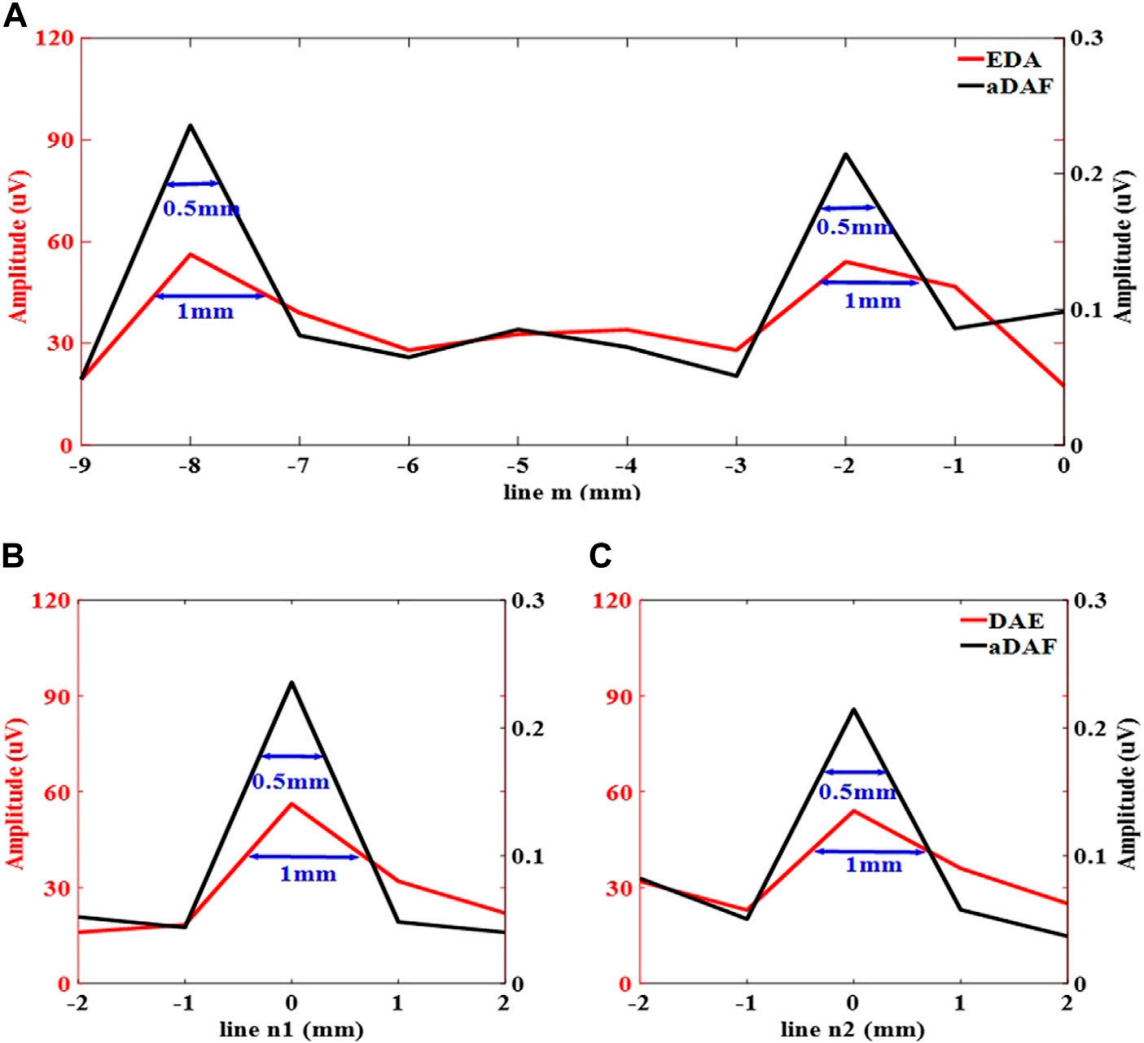
Line profiles of the decoded signal along the white dashed lines m, n1 and n2 marked in **(A)** along line m, **(B)** along line n1, **(C)** along line n2.

The 2-source imaging outcomes of employing the decoded AE signal are exhibited in Figure 7. The imaging with DAE is in Figure 6A, and Figure 6B shows the imaging with aDAF. It can be noticed that the two sources can be separated for both the imaging of DAE and aDAF. With the same dynamic range (5 dB), the imaging of aDAF can clearly locate the source position, while the imaging of DAE has artifacts. The SNR of the imaging adopting aDAF was 18.61 dB, in digits, and other has a SNR of 15.16 dB, the former is 22.75% higher than the latter.

The white dashed lines m, n1, and n2 indicated in Figure 6 were utilized to display the line profiles of the decoded signal in Figure 7. The sources positions in the *x* direction could be detected along line m at x = −8 mm and x = −2 mm with a strong signal. The position of sources in the *y* direction along lines n1 and n2 can be determined with a strong signal at y = 0 mm. With the −3 dB peak of the decoded signal, the source width of aDAF is 0.5 mm in both *x* and *y* directions. Additionally, the source width of DAE was 1 mm in both the *x* and *y* directions with the decoded signal of −3 dB peak. The imaging resolution of aDAF is 50% higher than that of DAE. In terms of the imaging resolution, then, aDAF is twice that of DAE.

#### 3.2.2 3-Source imaging result

**FIGURE 8:**
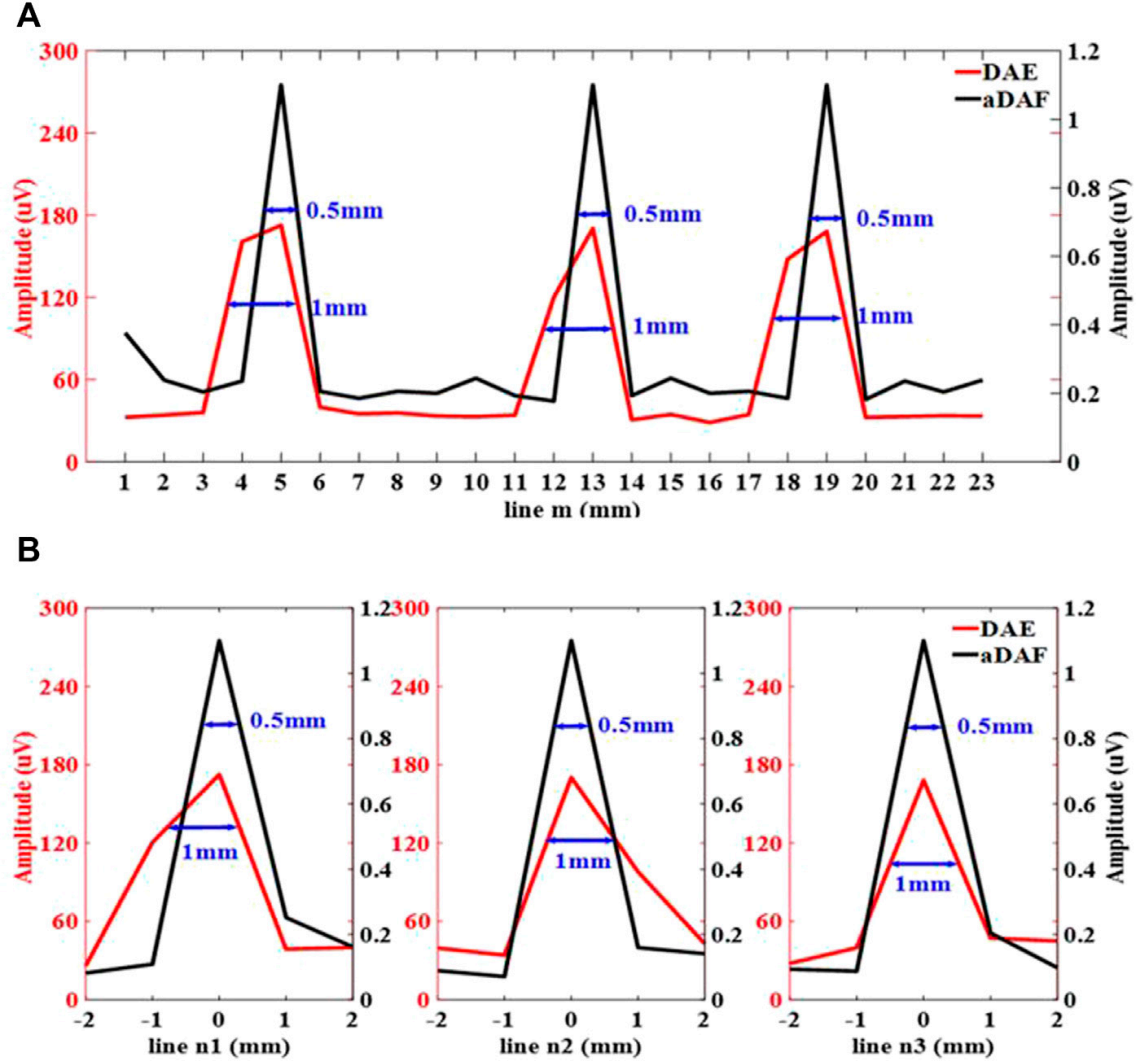
Line profiles of the decoded signal along the white dashed lines m, n1, n2 and n3 marked in **(A)** along line m, **(B)** along line n1, n2 and n3.

The imaging outcomes for the 3-source imaging experiment are displayed in Figure 8. As can be seen, for both DAE (Figure 8A) and aDAF (Figure 8B) images, all three sources can be clearly distinguished. Marked with blue circles, the SNR of the imaging with aDAF was 20.96 dB, which was greater than that with DAE of 16.92 dB. The SNR is boosted for the 2-source and 3-source imaging results by an average of 23.32%.

**FIGURE 9:**
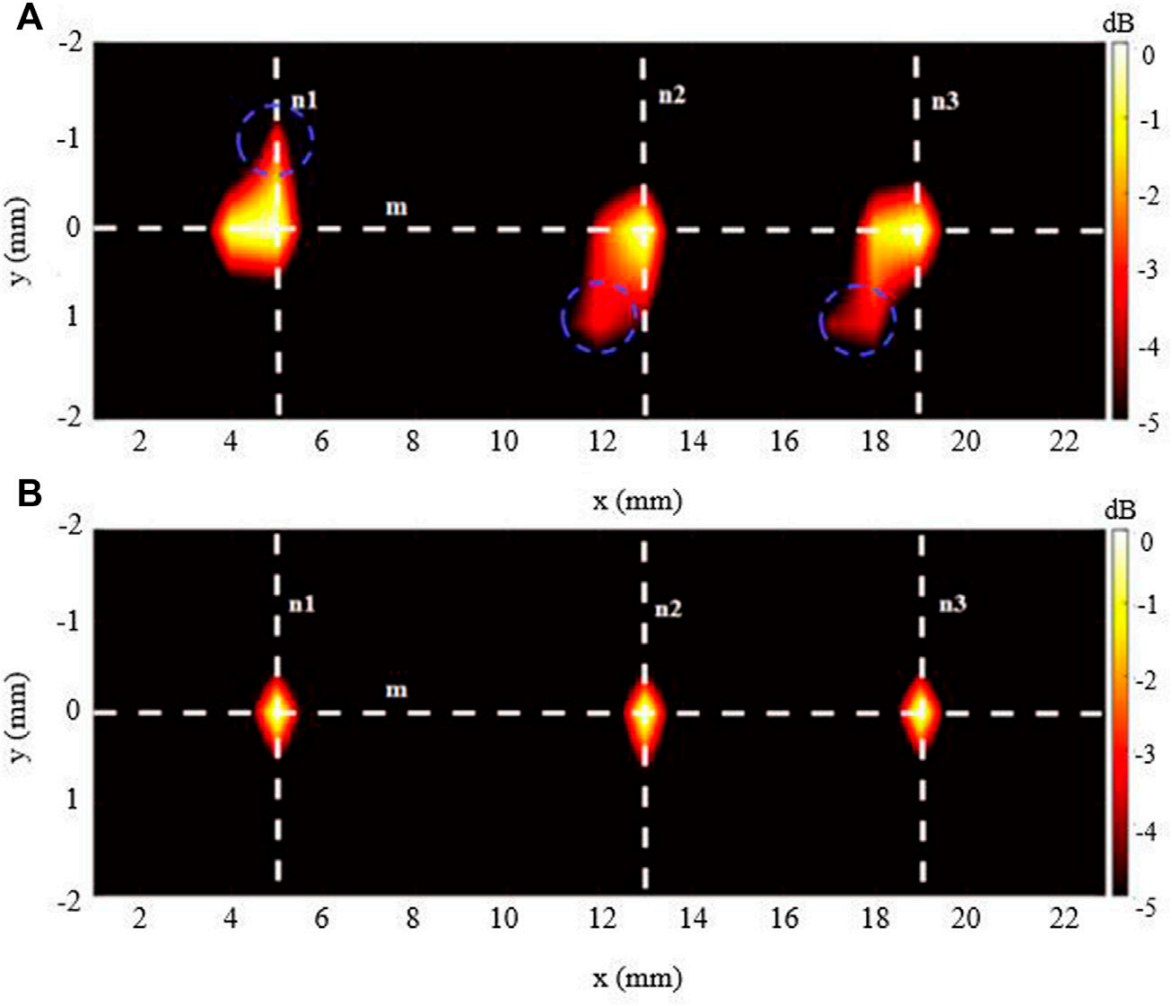
Imaging result of the 3-source with **(A)** DAE and **(B)** aDAF (hot colors, 5-dB dynamic range).

Figure 8 displays the line profiles of the decoded signal along the white dashed lines m, n1, n2 and n3 marked in Figure 9. Along line m, the sources positions in the *x* direction can be located with strong signals at x = 5 mm, x = 13 mm and x = 19 mm. Along lines n1, n2 and n3, the sources positions in the *y* direction can be located with a strong signal at y = 0 mm. With the −3 dB peak of the decoded signal, the source width of aDAF is 0.5 mm in both *x* and *y* direction. And, with the −3 dB peak of the decoded signal, the source width of DAE is 1 mm in both *x* and *y* directions. Compared with DAE, the imaging resolution of aDAF is also improved by 50%, which is consistent with the result of 2-source imaging.

## 4 Conclusion

The spatial resolution and decoding precision of ABI have a tangible improvement by applying the algorithm we suggested, giving the rise to advancements in the field of neural imaging. The greatest harmonic component adaptive selection and multiple harmonics decomposition of the AE signal envelope are also outstanding advantages of the aDAF, which is critical for applications in both clinical severe cases, especially for emergency-cases and brain computer interface of next-generation.

## Data Availability

The original contributions presented in the study are included in the article/supplementary material, further inquiries can be directed to the corresponding author.
